# Digital Technologies and Machine Learning in Environmental Hazard Monitoring: A Synthesis of Evidence for Floods, Air Pollution, Earthquakes, and Fires

**DOI:** 10.3390/s26030893

**Published:** 2026-01-29

**Authors:** Jacek Lukasz Wilk-Jakubowski, Artur Kuchcinski, Grzegorz Kazimierz Wilk-Jakubowski, Andrzej Palej, Lukasz Pawlik

**Affiliations:** 1 Department of Information Systems, Kielce University of Technology, 7 Tysiąclecia Państwa Polskiego Ave., 25-314 Kielce, Poland; lpawlik@tu.kielce.pl; 2 Institute of Internal Security, Old Polish University of Applied Sciences, 49 Ponurego Piwnika Str., 25-666 Kielce, Poland; grzegorzwilkjakubowski@wp.pl (G.K.W.-J.); andrzej.palej@o2.pl (A.P.); 3 Institute of Crisis Management and Computer Modelling, 28-100 Busko-Zdrój, Poland

**Keywords:** sustainable development, crisis management, hazard monitoring, floods, air pollution, earthquakes, fires, sensors, machine learning, image processing, Internet of Things

## Abstract

This review synthesizes the state of the art on the integration of digital technologies, particularly machine learning, the Internet of Things (IoT), and advanced image processing techniques, for enhanced hazard monitoring. Focusing on air pollution, earthquakes, floods, and fires, we analyze articles selected from Scopus published between 2015 and 2024. This study classifies the selected articles based on hazard type, digital technology application, geographical location, and research methodology. We assess the effectiveness of various approaches in improving the accuracy and efficiency of hazard detection, monitoring, and prediction. The review highlights the growing trend of leveraging multi-sensor data fusion, deep learning models, and IoT-enabled systems for real-time monitoring and early warning. Furthermore, we identify key challenges and future directions in the development of robust and scalable hazard monitoring systems, emphasizing the importance of data-driven solutions for sustainable environmental management and disaster resilience.

## 1. Introduction

In the context of the growing impact of climate change, increasing population density in vulnerable areas, and the intensification of extreme events, effective monitoring and prediction of natural and environmental hazards has become one of the major challenges facing contemporary engineering, Earth sciences, and information technology. Natural disasters such as earthquakes, floods, fires, and air pollution have serious consequences not only for public health, the environment and critical infrastructure, but also for the functioning of the economy and social security. Appropriate risk management, including both detection and rapid response, currently requires the integration of diverse data sources and the use of advanced analytical tools.

In recent years, there has been rapid development in digital technologies, which are significantly changing the approach to threat monitoring. Solutions based on the Internet of Things (IoT), machine learning (ML), deep learning (DL), and image processing techniques are becoming particularly important, as they enable the collection, analysis, and interpretation of data in real time, often with minimal human involvement. These technologies support the creation of early warning systems, threat forecasting, and decision-making in conditions of uncertainty.

**Figure 1:**
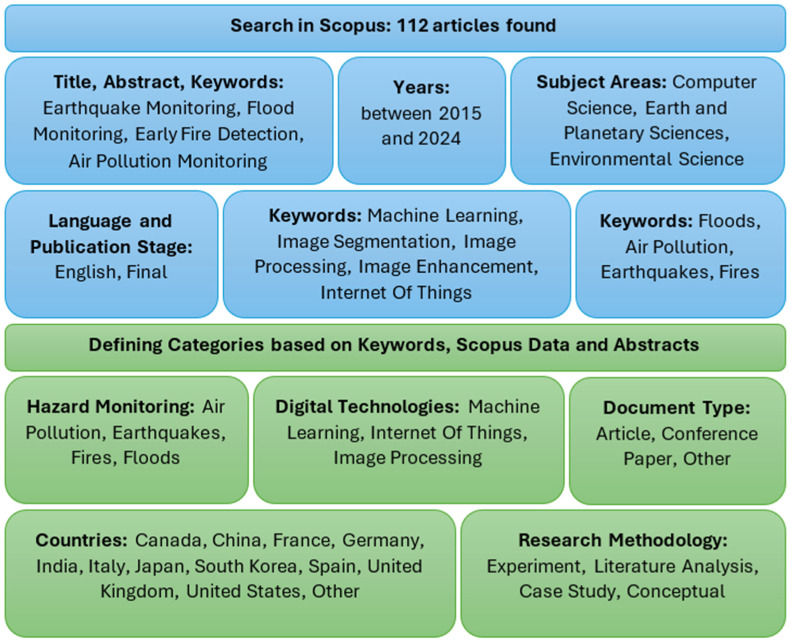
Method of qualifying publications for review analysis.

This article provides a comprehensive review of scientific research on the use of modern digital technologies in the process of monitoring environmental and natural hazards. The main objective of the study is to synthesize the state of the art by analyzing scientific publications from the SCOPUS database from 2015 to 2024, covering both review and research articles from recognized scientific journals and conference materials (detailed information on the methodology for qualifying publications is presented in Figure 1). In practice, this review focuses on four types of hazards, air pollution, earthquakes, floods, and fires, which are considered particularly important due to their frequency, their scope of impact, and the difficulty in detecting and counteracting their effects. Works presenting innovative approaches, practical implementations, and comparative studies of the effectiveness of various methods were taken into account. This made it possible to identify the main research trends, identify gaps, and indicate potential directions for the development of monitoring systems in the coming years.

The integration of digital technologies in the monitoring of natural and environmental hazards remains a relatively niche area of research. While scientific research contains numerous studies on individual types of hazards, such as floods, earthquakes, and fires, comprehensive approaches covering different types of phenomena and comparing the technologies applied are much rarer. There is also a lack of systematic studies that provide a synthetic overview of the state of the art, research gaps, and common challenges and opportunities for development in this area.

The main aim of this article is to systematize and fill this gap in the literature by reviewing scientific publications on the integration of digital technologies in monitoring four key categories of hazards: floods, air pollution, earthquakes, and fires. The analysis includes the identification of technological solutions used (including the Internet of Things, machine learning, satellite image analysis), data processing methods, ways of integrating multi-source information, and examples of implementations. The added value of this article is its holistic approach to the issue; the review covers four key categories of hazards mentioned earlier, analyzing them within a common research framework. This allows not only for a comparison of technologies and methods applied in different areas, but also for the identification of common elements and solutions that can be transferred between fields. In this sense, this article contributes a coherent classification of approaches to the literature, identifies research gaps, and formulates recommendations for future work, thus providing a reference point for researchers and practitioners interested in the development of modern hazard monitoring systems.

## 2. Materials and Methods

### 2.1. Data Selection

In practice, the selection process for publications on the subject of threat monitoring was based on a research procedure integrating elements of systems analysis. The detailed stages of this approach will be presented in the following sections of the article. At the initial stage, extensive bibliographic searches were conducted in the SCOPUS scientific database, aiming to identify works that met the established substantive and formal criteria. The search strategy involved combining predefined search terms with publication metadata, including titles, abstracts, and keywords.

To ensure the reproducibility of the systematic review, the literature search was conducted on 23 April 2025 using the Scopus database. The search strategy followed a two-stage refinement process:

1. Initial Search: The primary search query focused on hazard monitoring and digital technologies, restricted to English-language documents published between 2015 and 2024. The exact query string was as follows:

TITLE-ABS-KEY (“Earthquake monitoring” OR “Flood monitoring” OR “Early fire detection” OR “Air pollution monitoring”) AND PUBYEAR > 2014 AND PUBYEAR < 2025 AND (LIMIT-TO (PUBSTAGE, “final”)) AND (LIMIT-TO (LANGUAGE, “English”)) AND (LIMIT-TO (EXACTKEYWORD, “Machine Learning”) OR LIMIT-TO (EXACTKEYWORD, “Internet Of Things”) OR LIMIT-TO (EXACTKEYWORD, “Image Segmentation”) OR LIMIT-TO (EXACTKEYWORD, “Image Processing”) OR LIMIT-TO (EXACTKEYWORD, “Image Enhancement”))

Documents with types “dp” (Data Paper), “sh” (Short Survey), and “tb” (Table) were excluded, alongside irrelevant subject areas (e.g., Medicine, Arts, Pharmacology). This stage yielded 187 documents.

2. Refinement: The initial results were further filtered by focusing on the core keywords: air pollution, earthquakes, fires, and floods. This refinement resulted in a final dataset of 112 articles used for the qualitative synthesis and bibliometric analysis.

The results of this process made it possible to identify consistent thematic categories, which were then subjected to further analysis. Figure 1 presents a complete diagram of the data selection procedure applied.

The final analysis covered 112 scientific publications. The selection of material was based on metadata containing references to flood monitoring (floods), earthquakes (earthquakes), fire detection (fires), and air pollution monitoring (air pollution). In practice, English-language publications with final publication status, published between 2015 and 2024 in subject areas such as Earth and planetary sciences, environmental sciences, and computer science, were included. This makes it possible to ensure the reproducibility of the obtained data based on the applied methodological approach (a scientific novelty in the analyzed perspective).

The selection process used a set of keywords covering both technological terms—including machine learning, image segmentation, image processing, image enhancement, and Internet of Things technologies—as well as phrases describing the type of hazards: floods, air pollution, earthquakes, and fires.

Based on the collected material, four main groups categorizing the data were defined: (1) hazard monitoring, (2) digital technologies, (3) document type, and (4) research methodology. Classification of the 112 articles was performed manually based on the following operational definitions:

– Experiment: Studies involving primary data collection and controlled testing (e.g., laboratory testing of new low-cost gas sensors for air pollution monitoring).
– Case Study: Research focused on the implementation or demonstration of a system in a specific, real-world scenario (e.g., the implementation of a specific flood early warning system in a given river basin).
– Literature Analysis: Secondary research based on existing surveys, meta-analyses, or previously published data.
– Conceptual: Theoretical development of models (e.g., theoretical architecture of a deep learning model for seismic wave prediction without field validation) or systemic concepts without immediate field implementation.

The geographical classification includes countries where research was conducted, including China, the United States, India, the United Kingdom, Italy, Germany, France, Japan, South Korea, Spain, Canada, and other countries whose share was less than 3% of the total sample.

### 2.2. Data Analysis

**Figure 2:**
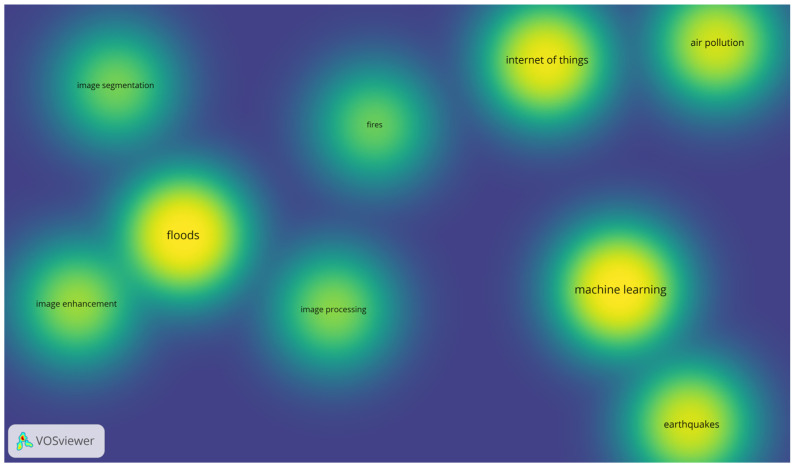
Identified hazard monitoring and digital technologies generated using VOSViewer software.

For the purposes of the bibliographic analysis, the authors’ methodological approach was applied. Moreover, in order to identify the dominant research directions, an analysis of the co-occurrence of keywords in publications from 2015 to 2024 was carried out using VOSviewer (v. 1.6.20) software [1,2]. The “density view” variant was used, in which the colors of the map reflect the intensity of research—from shades of blue (low intensity) through green to yellow (highest intensity), as shown in Figure 2. The brighter the area, the more often a given term appeared in combination with other keywords in the document set.

The density visualization presented in Figure 2 illustrates the concentration of research topics by calculating the density of keywords at any given point in the map. The intensity of the yellow color indicates a higher density of research focus, which is a function of both the number of items in the vicinity and their corresponding weights (Total Link Strength).

To ensure objectivity and reproducibility, the analysis was configured with a minimum keyword occurrence threshold of 10 and normalized using the Association Strength method. Furthermore, clustering was performed using the VOSviewer association strength-based clustering algorithm with a resolution parameter set to 1.0, resulting in the identification of three distinct thematic clusters. Table 1 provides the quantitative metrics for the core categories identified in the density hotspots.

As indicated by the data, while “Floods” remains the most established topic (highest TLS of 50), the recent “hotspots” in the density map for “Fires” and “Deep Learning” (Avg. Pub. Year > 2022) suggest an emerging shift in the research landscape towards more advanced computational methods and previously less represented hazard types.

The most prominent cluster is formed by the keyword “floods”, located in the center of the map and marked in yellow. The high density of this node indicates that flood research is a key focus of the analyzed literature corpus and is strongly linked to image processing terms (including image segmentation, image enhancement, and image processing). This suggests that the detection and forecasting of hydrological phenomena is largely based on visual analysis methods (satellite images, aerial photographs, drone data).

Two further clusters stand out in the lower part of the visualization. The first one includes the term “machine learning”, also with high intensity (yellow color), which confirms the significant role of machine learning methods in environmental research. Right next to it is “earthquakes”, which indicates the frequent combination of ML algorithms with seismic data analysis, e.g., in early warning systems or earthquake classification.

The combination of the technological and environmental terms “Internet of Things” and “air pollution” leads to the conclusion that air quality research is increasingly based on distributed networks of IoT sensors, enabling real-time measurement of pollutant concentrations.

A less concentrated but still significant area concerns the keyword “fires,” located between technology clusters and the group of natural hazards. The relatively lower intensity indicates that the issue of fires is moderately represented in the literature and is not yet permanently associated with one leading technology; rather, there is scattered use of both visual techniques and IoT sensors or machine learning methods. The resulting picture indicates the main research axes:

− Visual analysis of floods (image processing dominance);
− Machine learning in seismology (earthquakes);
− Integration of IoT with air pollution monitoring.

The relatively weaker representation of the topic of fires—coupled with the lack of a clear link to specific technology—highlights a research gap that needs to be filled by future interdisciplinary projects.

Although Figure 2 shows the aggregated distribution of keywords to ensure comparability between the types of hazards analyzed, the literature review also reveals characteristic clusters of concepts specific to each category. Research on floods focuses primarily on hydrological modeling, remote sensing, and spatial data analysis. In the area of air pollution, keywords related to sensor networks, real-time monitoring, and health exposure assessment dominate. Work on earthquakes focuses mainly on seismic signal processing, early warning systems, and anomaly analysis, while research on fires emphasizes the role of image processing, satellite data, and temporal–spatial pattern recognition.

In practice, the Chi-square test (χ^2^) was applied in the analysis to assess the relationship between qualitative variables. The aim of the procedure is to compare the observed values with the expected values, assuming the independence of the variables. The χ^2^ statistic, calculated on the basis of these differences, is interpreted in relation to the Chi-square distribution with the appropriate number of degrees of freedom, depending on the number of categories of the analyzed variables. The obtained *p*-value allows the statistical significance of the result to be determined; if *p* < α (α = 0.05), a significant relationship between the variables under study is assumed to exist.

It should be noted that for some hazard categories, particularly fires in the 2015–2019 period, the number of publications was very small. As a result, the statistical power of the Chi-square test is limited, and non-significant results should not be interpreted as evidence of the absence of meaningful differences.

To limit bias resulting from the overall increase in the number of publications over time, comparisons between the periods 2015–2019 and 2020–2024 were based on the structure of distributions (shares) of categories within the corpus, rather than on raw counts. A Chi-square test was used to assess whether the composition of publications (e.g., by technology type, application scenario, or research method) differed between periods after accounting for differences in the total number of papers.

## 3. Quantitative and Qualitative Analysis of the State of the Art

In recent years, there has been a steady increase in the number of scientific publications devoted to monitoring threats using digital technologies. As mentioned earlier, an analysis of publications from 2015 to 2024 covered four categories of threats:

1. Floods;
2. Air pollution;
3. Earthquakes;
4. Fires.

The growing interest in these topics reflects the increasing need to develop effective early warning systems and precise threat monitoring, which is possible thanks to advances in sensors, data analytics, and communication technologies.

In order to move from a simple literature review to an in-depth synthesis, the following sections have been developed based on a thematic and comparative synthesis. Publications are not discussed individually, but grouped into coherent research clusters representing recurring trends in technology development, analytical methods, and practical applications. Each group compares approaches in terms of their methodological assumptions, effectiveness, and limitations, allowing us to capture dominant patterns and the evolution of research paradigms. This structure not only allows us to identify trends, but also to critically assess the maturity of technologies and their implementation potential in the context of threat monitoring.

As a result, Section 3.1, Section 3.2, Section 3.3 and Section 3.4 do not constitute a collection of individual descriptions, but rather an integrated research narrative showing the interrelationships, complementarity, and limitations of the analyzed methods.

An analysis of the collected studies allows for the identification of recurring relationships between the digital technologies used and the specific nature of the threats being monitored. These relationships indicate that the selection of analytical tools is strongly linked to the nature of the data and the monitoring objectives in specific research areas.

In the next part of the paper, a synthetic review of the literature is presented, focusing on the main research directions, technologies used, and monitoring objectives for each of the identified threat categories.

Section 3.1, Section 3.2, Section 3.3 and Section 3.4 have been deliberately designed in accordance with the standardized evidence mapping convention (data–method–purpose) to ensure a transparent and replicable link between each publication and the categories of analysis.

### 3.1. Floods

An analysis of the number of publications between 2015 and 2024 indicates that floods were the most frequently discussed issue in the area of hazard monitoring. A total of 43 articles on this topic were identified (12 between 2015 and 2019, 31 between 2020 and 2024). The dominance of this research area suggests that the problem of flooding is perceived as one of the most serious environmental challenges, requiring continuous improvement in detection and forecasting methods.

In Ref. [3], a state-of-the-art IoT and deep learning-based water level measurement method using Mask Region-Based Convolutional Neural Network (Mask-RCNN) for precise image analysis is presented. The proposed solution eliminates the limitations of traditional sensors, such as vulnerability or inefficiency at night, by providing automatic, fast, and low-cost monitoring. The authors note that the technology has significant potential in water resource management and flood prevention systems. In Ref. [4], the authors show that, through bistatic coherence analysis and image segmentation, it is possible to produce an accurate binary map of water vs. non-water and classify water as permanent or temporary. This product, with high coherence (up to 93% in Europe), represents a valuable tool for scientific research and will be made publicly available. Ref. [5] presents a new preprocessing method for training samples for remote sensing image classification based on local statistics and quadrant partitioning. This technique, supported by post-processing filters, improves classification accuracy, particularly when using Support Vector Machine (SVM) classifiers. In Ref. [6], a multi-step approach for flood detection using Chinese satellite data of various types (synthetic aperture radar, multispectral, and hyperspectral) is presented, combining pre-segmentation with deep learning based on noisy labels. The method has proven effective and fast in accurately monitoring floods, as demonstrated by the example of Poyang Lake. In Ref. [7], an automatic system for detecting and estimating the depth of urban floods based on images and video recordings, without the need for traditional sensors, is proposed. It combines image segmentation with reference object analysis, achieving high accuracy and reliability even in difficult weather conditions. A new SOWI (Optical Imagery Water Index), combining optical and radar data for precise surface water extraction, is demonstrated in Ref. [8]. The method improves accuracy compared to single-sensor approaches, reaching efficiencies of up to 99%, and proves effective in monitoring floods, including in Henan Province in 2021. In Ref. [9], an analysis was conducted on the use of the Normalized Difference Water Index (NDWI) and Modified Normalized Difference Water Index (MNDWI) to identify flooded areas in the Tarwin catchment in Australia based on Landsat-8 data. The NDWI proves more effective, achieving an accuracy of 96.04%, and the study also reveals the limited effectiveness of local dikes. The effectiveness of flood detection using PALSAR-2 radar data during the 2015 floods in Japan was analyzed in Ref. [10]. The study shows that optimal observation conditions allow for fast and effective flood monitoring, even in built-up areas. In Ref. [11], two Intelligent Data Models for the integration and analysis of diverse satellite data and flood risk assessment are proposed to support efficient water data management using artificial intelligence technologies, which improve the performance of flood monitoring in practice. Ref. [12] presents a methodology for creating an integrated information system combining IoT and GIS (Geographic Information System) technologies for regional flood monitoring, which significantly improves forecasting and decision support. In Ref. [13], a Bayes_Opt-SWMM tool for calibration and uncertainty optimization in real-time urban flood modeling using Bayesian optimization and a Gaussian process-based surrogate model is presented. Ref. [14] shows a method for detecting and quantifying flood water levels from VGI (Volunteered Geographic Information) images taken with smartphones using random forest classification and photogrammetry with drone-derived elevation models. In Ref. [15], a new adaptive data fusion algorithm was developed, combining high-resolution MODIS (Moderate Resolution Imaging Spectroradiometer) data and cloud-tolerant microwave data to accurately predict daily land surface water coverage. In Ref. [16], a method combining SAR (Synthetic Aperture Radar) data with shallow water modeling for local flood risk estimation is presented that improves the accuracy of determining flood extent and return periods in sub-catchments. It should be noted that tests on the River Severn catchment have confirmed the effectiveness of the approach in monitoring flooding at a local level. In Ref. [17], the drivers of flooding were assessed, and the effectiveness of four machine learning models for predicting and mapping flood vulnerability in the Amibara district, Ethiopia, is compared. In Ref. [18], Sentinel-1 radar data and machine learning algorithms, including CNNs (Convolutional Neural Networks), were applied to map and monitor floodplains, particularly rice fields, in the Mekong Delta, An Giang Province. In Ref. [19], the use of IoT technology for intelligent environmental monitoring, including local meteorological conditions, water levels, soil moisture, air quality, and earthquake detection, is demonstrated. In Ref. [20], a method using a Convolutional Neural Network to accurately and rapidly monitor flooding from multispectral satellite data is presented, effectively minimizing the influence of clouds and other disturbances. In Ref. [21], flow in the Yangtze River basin is estimated by combining altimetry satellite data with hybrid models and machine learning. In Ref. [22], a program using OCR (Optical Character Recognition) techniques is developed to automatically recognize the date and time in radar images of rainfall in Thailand. In Ref. [23], it was shown that a single well-timed SAR image can significantly improve forecasts over extensive, flat floodplains, whereas narrow valleys require more images for efficient and cost-effective flood monitoring. In Ref. [24], a fully automated flood monitoring system based on deep learning running on a cloud platform is demonstrated. In Ref. [25], a multi-temporal approach for classifying freshwater macrophytic vegetation types in the floodplain of the lower Paraná River is analyzed using seven COSMO-SkyMed X-band radar images along with segmentation and classification algorithms. In Ref. [26], a method combining deep learning, computer vision, and crowdsourcing to detect and estimate flood depths from smartphone photos in flooded areas is shown. In Ref. [27], a new reservoir extraction model based on deep neural U-Net is presented, integrating satellite data with additional geospatial layers to improve segmentation accuracy for flood monitoring in Southeast Asia. In Ref. [28], the use of satellite imagery and the R2CNN algorithm was proposed for mapping and monitoring flash floods, addressing the limitations of traditional surveillance methods. In Ref. [29], researchers used the ELECTRE (Elimination and Choice Expressing Reality) multi-criteria decision-making technique to select optimal spectral bands of Sentinel-2 satellite imagery, enabling more accurate and faster detection of flood-affected areas. In Ref. [30], a system applying the DeepLabv3 network is demonstrated for automatic detection and segmentation of flooded areas in satellite imagery with 87% accuracy, and the integration of satellite data with IoT and meteorological forecasts is shown to enable effective early warning and flood management. In Ref. [31], an automated method for estimating flood duration and its uncertainty based on multi-temporal flood extent masks from Sentinel-1, Sentinel-2, Landsat-8, and TerraSAR-X satellite data is presented. This method, which operates in near real time, has been successfully tested on floods in Mozambique and Bihar, supporting emergency mapping activities. In Ref. [32], researchers present a fully automated rapid flood mapping method based on CNNs using radar data, which reduces mapping time by 80% while maintaining high efficiency and integration with continuous monitoring systems. In Ref. [33], floods in Weifang (China, 2018) were monitored using full polarization data from Sentinel-1A and Doublet, Otsu, and Region Growing methods to detect water bodies in radar images. In Ref. [34], a method developed by Dares Technology to detect floods by applying SAR imagery for more effective emergency response without the need for training data is presented, validated using the 2017 Mumbai floods as an example. In Ref. [35], an integrated methodology for assessing the impact of the 2022 Pakistan floods on cultural heritage is shown, combining Sentinel satellite data, field information, and interdisciplinary collaboration to improve risk management and conservation. In Ref. [36], the application of a machine learning algorithm and object-based classification on SAR data for effective flood monitoring is analyzed, confirming the usefulness of Sentinel-1 imagery in flood risk reduction. In Ref. [37], an effective method for detecting flooded urban areas using a modified deep learning network is shown which copes with shadows and limited training data to improve the accuracy of flood mapping from aerial imagery. In Ref. [38], SAR Sentinel-1 imagery and random forest classification are applied for flood mapping and damage assessment in three Indian cities, demonstrating their usefulness in rescue operations due to all-weather capability. In Ref. [39], an IoT-FSP (Internet of Things-based Flood Status Prediction) model is shown, using IoT and machine learning algorithms to achieve high-accuracy flood status predictions and early warning. In Ref. [40], a probabilistic surface water segmentation method based on computer vision is proposed, which effectively maps flooded areas with limited training, achieving results comparable to classical methods during floods in France. In Ref. [41], an effective water segmentation method using SAR imagery from KOMPSAT-5 (KOrean Multi-Purpose SATellite) and deep learning models is presented, with the highest accuracy obtained using HRNet (High-Resolution Network), enabling precise flood monitoring regardless of weather conditions. In Ref. [42], a flood early warning system based on sensors and machine learning algorithms is described, effectively classifying water hazard levels and sending real-time alerts with up to 99.5% accuracy using random forest (RF). In Ref. [43], an automated particle velocity visualization and topography reconstruction system was developed for monitoring and analyzing flash floods in mountain streams. In Ref. [44], the development and testing of an efficient flood monitoring platform is analyzed, allowing for simultaneous collection and management of data from multiple sensors with low processor consumption. Finally, in Ref. [45], the use of Landsat TM/ETM+ (Thematic Mapper/Enhanced Thematic Mapper Plus) satellite data for high-frequency spatial and temporal monitoring of snowmelt floods in Siberia was presented, demonstrating its usefulness despite weather and temporal constraints.

An analysis of 43 papers showed that research on flood monitoring focuses primarily on the integration of modern digital technologies—in particular satellite imagery (SAR, optical, hyperspectral), the IoT and GISs, deep learning methods, and advanced photogrammetry. A large proportion of the publications present proprietary algorithms and indicators that increase the accuracy of water detection, classification of flooded areas, and flood forecasting. The main research areas covered the following categories presented in Table 2.

An analysis of 43 publications on flood monitoring indicates a clear shift towards multi-source approaches (fusion of satellite, in situ/IoT, and meteorological data), which increases the resilience of systems to data gaps and extreme conditions. At the same time, the importance of deep learning methods in the detection and segmentation of flood areas is growing, although their effectiveness remains highly dependent on the quality of training data and local conditions (transferability between regions). From an implementation perspective, near-real-time solutions are becoming crucial, but there are limitations in terms of infrastructure costs, pipeline integration, and the sensitivity of models to data shift. The most commonly identified gaps include the lack of standardized benchmarks and validation protocols, limited assessment of model generalizability, and insufficient reporting of operationally relevant metrics (e.g., false alarms, response time).

In the context of remote sensing, in addition to satellite data, it is also worth considering radar systems, including high-frequency ocean radar, which can provide high temporal resolution observations and serve as a complementary source for monitoring hydrological hazards in coastal areas and estuaries.

Finally, it is also worth noting that graph neural networks (GNNs) may also play an increasingly important role in flood applications, especially when the data have a network structure (e.g., sensor layout, river network), as they enable the modeling of spatial dependencies and relationships between nodes and can be useful for multi-source data fusion.

The automation of detection and analysis processes shortens response times and increases the effectiveness of early warning systems. Many studies emphasize the need to integrate multiple data sources—both from ground-based and satellite sensors—to increase the accuracy and resilience of systems to interference. Real-time mapping is of great importance, as it is crucial for crisis and evacuation operations.

The review of publications also shows that flood monitoring is an area where the synergy between digital technologies, big data processing, and crisis management systems is most evident.

### 3.2. Air Pollution

The second most frequently published topic was air pollution, with 32 articles (8 in 2015–2019 and 24 in 2020–2024). The increase in interest in this issue in recent years is linked to growing awareness of the health effects and the need to introduce tools for continuous air quality monitoring.

In Ref. [46], the authors describe the development and implementation of a wireless sensor network with advanced machine learning algorithms to monitor and rapidly reduce urban air pollution, thereby improving air quality and supporting sustainable urban development. In Ref. [47], the effectiveness of different machine learning algorithms in detecting air pollutants using data from the Open Government Data India platform was compared, analyzing their accuracy in monitoring harmful substances. Ref. [48] verifies the usefulness of satellite-based NO_2_ data from Sentinel-5P in air pollution monitoring in Poland, showing that a random forest model accurately estimates surface NO_2_ concentrations and identifies key influencing factors. In Ref. [49], the feasibility of using Landsat imagery and machine learning algorithms to produce high-resolution (30 m) optical aerosol depth estimates was investigated, achieving high accuracy and potential for local applications in air pollution monitoring. In Ref. [50], a method for reconstructing air pollution sensor network data based on graphs learned from measurements was proposed, which effectively fills in missing data, achieves virtual detection and data fusion, and improves monitoring accuracy in heterogeneous sensor systems. In Ref. [51], an IoT-based real-time air pollution monitoring system is demonstrated to collect, analyze, and visualize air quality data in different urban zones, storing such data in the cloud and facilitating effective environmental management. In Ref. [52], an efficient method for filling in missing data in IoT sensor networks for environmental monitoring based on k-means clustering is presented, which improves the accuracy of sensor calibration compared to other techniques, despite higher computational complexity. In Ref. [53], advanced methods for sequential simulation of high-resolution spatial distributions of air pollutants (PM2.5, SO_2_, ozone) in China using an optimized LightGBM (Light Gradient Boosting Machine) model are presented, which improve the accuracy and stability of predictions and are of great importance for population health monitoring and assessment. In Ref. [54], the calibration of a low-cost PM2.5 monitor using an exposure experiment and a machine learning (random forest) model was described, which showed better accuracy than linear regression, allowing the low-cost monitors to be used more effectively in future air pollution and health research. In Ref. [55], methods for reconstructing missing data in heterogeneous air pollution monitoring networks are compared, showing that a technique based on signal smoothness together with Laplacian interpolation provides high accuracy and network robustness to sensor failures, outperforming traditional distance-based and linear regression methods. Ref. [56] presents a systematic review of the use of UAVs (Unmanned Aerial Vehicles) for air quality and odor monitoring at wastewater treatment plants, pointing out the lack of dedicated solutions and proposing an integrated UAV system equipped with gas sensors, multispectral cameras, and thermal imaging that can improve the management, safety, and operational efficiency of wastewater treatment plants. In Ref. [57], the authors proposed a multi-component network (Multi-AP learning network) for spatial and temporal estimation of pixel-level concentrations of multiple air pollutants based on station measurements and urban data, which allows for efficient production of accurate, high-resolution pollution maps with fewer measurement points and significantly lower computational cost. Ref. [58] presents a comprehensive overview of APMSs (Air Pollution Monitoring Systems), analyzing their technologies, protocols, and performance and discussing the prospects for developing more realistic, scalable, and accurate solutions based on wireless sensor networks and IoT. Ref. [59] proposes the design of an IoT-based air pollution monitoring system that, through integrated sensors, a microcontroller, a communication module, and software, provides real-time data collection, processing, analysis, and visualization, supporting rapid response to exceedances of air quality standards and promoting environmental protection measures. In Ref. [60], a novel approach to air pollution forecasting is presented that uses camera images enhanced with weather data and generative antagonistic networks and data augmentation, achieving high accuracy in pollution estimation comparable to traditional models based on historical data. Ref. [61] describes the development and implementation of a low-cost IoT-based remote air pollution monitoring system using Arduino and Raspberry Pi modules and gas sensors that transmits data to the cloud in real time, providing accurate and accessible air quality monitoring. In Ref. [62], the implementation of a monitoring system for odor emissions using a landfill example is characterized, applying a wireless sensor network with an electronic nose. The authors highlight that this approach improves the cost-effectiveness of monitoring and enables rapid response to nuisance odor emissions, meeting the requirements of specific air pollution control programs. Ref. [63] analyzes the challenges and benefits of using cyber–physical–social systems for air pollution monitoring in smart cities, highlighting their potential to overcome the limitations of traditional methods and to engage citizens. In Ref. [64], a machine learning approach (CatBoost) was proposed to reconstruct missing air pollution data from neighboring sites, allowing a consistent long-term (2006–2022) dataset to be established in the Pearl River Delta and demonstrating trends in changes in concentrations of major pollutants, highlighting the importance of continuous monitoring for air quality protection. Ref. [65] presents a graph signal reconstruction framework for improving data quality in air pollution monitoring networks, using a graph learned from the data and comparing different signal reconstruction methods on real measurements, showing the advantage of kernel-based methods and proposing scalable solutions for large sensor networks by partitioning the network with clustering algorithms. Ref. [66] describes the design of an IoT-based intelligent air pollution monitoring system that collects data from various sensors at urban intersections, visualizes it on a smart mirror, and uses an advanced machine learning model to predict PM10 levels, supporting traffic management and environmental control to improve air quality in congested cities such as New Delhi. Ref. [67] presents an innovative approach to monitoring urban air quality through visual analysis of haze in long-range images, using the new fastDBCP (fast Dark and Bright Channel Prior) method, which effectively correlates haze indices with actual pollution levels, offering a fast and accurate tool for assessing air conditions. In Ref. [68], a multimodal method for organizing and visualizing spatial–temporal Big Data from IoT networks, embedded in Twin Virtual Geographic Environments, is presented, which enables dynamic monitoring and analysis of air pollution in urban industrial parks and supports early warning and decision-making through advanced data analysis and 3D (three-dimensional) modeling. Ref. [69] proposes a hybrid method for calibrating global PM2.5 data products using machine learning models that significantly improves the accuracy of their measurements, reducing errors and providing high-quality, continuous data for global air pollution monitoring and health studies. In Ref. [70], a two-step strategy for the validation and calibration of low-cost ozone sensors in an urban air pollution monitoring network is presented, highlighting the post-implementation challenges and the need for a cyclic, continuous calibration approach that reduces reliance on reference instruments and improves measurement accuracy over time. In Ref. [71], six advanced machine learning models were developed and evaluated for predicting the daily Air Quality Index (AQI) in Indian cities. In Ref. [72], energy-efficient smart IoT devices have been developed for monitoring air pollution and energy consumption that use 5G (fifth-generation) connectivity, local data processing, and compression techniques to optimize power consumption, memory, and network bandwidth. In Ref. [73], a data aggregation mechanism based on compressive sampling with auxiliary information is presented that improves the quality of data reconstruction in smart city air pollution monitoring using IoT devices. In Ref. [74], a concept for an IoT-based interactive online system for air pollution monitoring and health risk assessment in urban congestion using traffic and weather data was developed. In Ref. [75], a microcontroller-based toxic gas detection, alarm, and clean-up system is presented that can significantly enhance safety in public and private spaces, supporting the development of smart cities. In Ref. [76], a trust model for data sharing in smart cities was developed that enhances transparency and accountability through semantic technology and data use rules, using air pollution monitoring as an example. Finally, Ref. [77] describes an air pollution monitoring and forecasting system based on IoT technology and neural networks, which enables accurate real-time measurements and forecasts due to its low cost and scalability.

An analysis of 32 scientific articles showed that research on air quality monitoring focuses on the integration of IoT technologies, wireless sensor networks, satellite remote sensing, and advanced machine learning methods. There is a clear trend towards low-cost, scalable, real-time systems that also ensure high measurement accuracy. The main research areas are listed in Table 3.

The analyzed literature on air pollution monitoring shows the complementarity of technological approaches, resulting from a compromise between accuracy, cost, and temporal and spatial resolution. Ground-based measurements (reference stations and IoT/WSN networks) provide high temporal resolution but limited spatial coverage; in the case of low-cost sensors, calibration supported by ML methods is crucial. Satellite data offer wide area coverage, but have limited temporal resolution and require validation and downscaling to ground-level concentrations. Mobile platforms (e.g., UAVs) enable very detailed local measurements, but are costly and more difficult to scale. As a result, the most useful solutions are those that integrate multiple data sources, improving temporal and spatial consistency and reducing uncertainty at the cost of greater implementation complexity.

If we summarize, based on the analysis conducted, it can be concluded that the importance of reconstructing missing data and calibrating sensors is emphasized, which is crucial for the quality of measurements in large, heterogeneous networks. Studies are increasingly using techniques for merging data from multiple sources (ground sensors, satellite data, cameras, weather data) and multimodal solutions that integrate different types of information within a single system.

Innovative methods of data collection are also being developed, such as visual analysis of haze in photographs, the use of unmanned aerial vehicles, and electronic ‘noses’ for odor detection. Air quality monitoring is increasingly linked to predictive and early warning systems, supporting environmental protection and public health policies.

### 3.3. Earthquakes

The third most frequently addressed issue in the analyzed literature was earthquakes, which constitute an important area of research in the field of natural hazard monitoring. Between 2015 and 2024, a total of 26 articles were published, the vast majority of which (22) were written in the last five years, indicating a growing interest in this topic.

In Ref. [78], a weakly supervised DAS-N2N (Distributed Acoustic Sensing—Noise2Noise) machine learning method was proposed to effectively suppress noise in distributed acoustic sensor data without the need for labeling, allowing for more accurate detection of microseismic events. In Ref. [79], the authors analyze a seismic sequence in the Apennines in Italy, showing that even moderate earthquakes can activate extensive fault segments, and that machine learning methods allow for fast and accurate monitoring of these processes for short-term predictions. In Ref. [80], a new deep learning model is presented that effectively detects P and S seismic waves in regional data from Northern Europe, outperforming existing methods in terms of precision and accuracy, especially in continuous processing mode. In Ref. [81], a state-of-the-art U-Net algorithm based on neural networks and data augmentation is analyzed, which allows for more accurate and efficient real-time monitoring of earthquakes, even under high-seismicity conditions, as shown in California. In Ref. [82], an interferometric backscatter imaging method is described that improves the accuracy and stability of microseismicity localization by suppressing random fluctuations in reconstructed wavefields arising from sparse data sampling or velocity model uncertainties. Ref. [83] shows the application of deep learning at the U.S. Geological Survey National Earthquake Information Center to improve automatic seismic phase detection and classification, which enhances the accuracy of determining wave arrival times, phase types, and source-to-station distances, and consequently increases phase association and earthquake location and reduces false detections. In Ref. [84], an ArrayConvNet Convolutional Neural Network is described, which directly analyzes continuous seismic network data, enabling accurate and efficient earthquake detection and location with high sensitivity and low computational cost, which significantly outperforms traditional methods and is particularly suitable for real-time monitoring. In Ref. [19], it is shown how IoT technology together with machine learning and modern sensors transforms traditional environmental monitoring into an advanced intelligent system, enabling efficient tracking of air quality, weather, soil moisture, and seismic activity, which supports better management and faster response to environmental hazards. In Ref. [85], it was pointed out how the MALMI (MAchine Learning-aided earthquake MIgration location) workflow process combines machine learning with the wavelet migration method to enable automatic, accurate detection and location of microseismic events with a low signal-to-noise ratio in real time, which allows more seismic events to be detected compared to traditional methods and is available as an open-source tool. In Ref. [86], a machine learning method is presented that emulates seismic wave transition times calculated from an accurate global 3D model of the Earth, offering a fast and compact approximation with very low error (about 0.1–0.2 s), comparable to traditional ray-tracing calculations, enabling more efficient seismic event localization. In Ref. [87], a low-energy, high-precision earthquake monitoring system installed in buildings was developed and evaluated. The system records vibrations in real time and transmits data via LoRa (Long-Range) networks to the cloud to assess structural resilience and support the design of more robust buildings against seismic shaking. In Ref. [88], OBSTransformer (Ocean-Bottom Seismometer Transformer), a generalized seismic phase detector for ocean-bottom seismometers, was introduced. Through automatic data labeling and transfer learning on a large global dataset, it substantially enhances the detection of P- and S-phases, particularly over long distances, and demonstrates higher performance and noise robustness compared to previous models trained on land-based seismic data. Ref. [89] describes how the Mw 5.0 earthquake that struck Qiaojia, China, during the filling of the Baihetan reservoir was examined using machine learning techniques and advanced localization methods. In Ref. [90], CubeNet, a generalized neural network based on a 3D U-Net architecture, was proposed to incorporate the spatial correlation of signals from multiple seismic stations, thereby increasing the accuracy of P- and S-wave phase identification and effectively handling irregular station distributions and impulsive noise, making it particularly suitable for analyzing large-N seismic network data. Ref. [91] presents an open-source Python 3.12.2 package for rapid seismic data processing using deep learning, providing advanced models for earthquake detection, seismic phase picking, polarization analysis, and magnitude estimation that surpass existing approaches in both accuracy and efficiency. In Ref. [92], a model for seismic phase identification on ocean-bottom seismometer data was developed using transfer learning with a limited training set; it achieves higher accuracy and smaller arrival time errors—especially for P-waves—outperforming four existing deep learning models. In Ref. [93], an intelligent earthquake monitoring and prediction framework based on collaboration, IoT, and fog and cloud computing is proposed. Through data preprocessing and the use of random forests and the Adaptive Neuro-Fuzzy Inference System (ANFIS), it effectively forecasts event magnitudes while maintaining low latency and high accuracy suitable for real-time monitoring. In Ref. [94], a method for rapid, location-independent estimation of earthquake magnitude from raw single-station P-wave waveforms was introduced using the regression model MagNet. In Ref. [95], seismicity induced by fluid injection in the Brady Hot Springs geothermal field (Nevada) was tracked using an 8.6 km DAS cable and a neural network called ADE-Net2, identifying 90 earthquakes, including 21 previously unrecognized events. In Ref. [96], a CNN ConvNetQuake_INGV network was proposed that, based on 50 s signals from a single station, can detect and probabilistically characterize earthquakes at different distances, achieving 87% detection accuracy. In Ref. [97], a fast and reliable method for the comprehensive estimation of earthquake magnitudes from raw waveforms recorded at single stations is presented. In Ref. [98], a combination of an empirical fitted field method and Convolutional Neural Networks was shown to more effectively detect and classify iceberg calving events on Svalbard, improving monitoring of ice loss and understanding of climate change in the region. In Ref. [99], QuakeFlow is presented as a scalable, cloud-based workflow that employs deep learning to rapidly and accurately detect, locate, and estimate earthquake magnitudes from large seismic datasets, markedly increasing the number of detected events and enabling real-time monitoring. In Ref. [100], MagNet (Magnitude Neural Network) is described in light of research—a deep learning algorithm for automatic and stable estimation of earthquake magnitudes based on complete wave records from seismic networks—which shows high accuracy for small and moderate events and copes with disturbances, although it requires further data to improve estimates of large earthquakes. Ref. [101] presents a scalable and portable Agent Processing Platform that uses multi-agent systems to monitor earthquakes through a distributed learning algorithm on data from heterogeneous seismic and geodetic sensor networks, with the possibility of extension to common mobile devices such as smartphones. In Ref. [102], an ULF (Ultra-Low Frequency) electric field classifier based on a backpropagation neural network was designed, which, based on the statistical characteristics of signals recorded during the Wenchuan earthquake, effectively classifies data with 72.3% accuracy.

An analysis of 26 scientific articles has shown that earthquake research is increasingly focusing on the use of deep learning, machine learning, and distributed measurement systems, enabling real-time detection, localization, and magnitude assessment of events. In recent years, there has been a dynamic development of neural network algorithms (CNN, U-Net, OBSTransformer, CubeNet, MagNet), which outperform traditional methods in terms of precision, noise resistance, and the ability to work with large datasets. The research areas addressed are presented in Table 4.

Based on the review, it can be concluded that a significant part of the research focuses on microseismic detection and early warning systems using data from extensive seismic networks, bottom seismometers, distributed acoustic sensing systems, and sensors installed in buildings. The integration of multiple data sources—seismic, geodetic, and environmental—is often used to increase the effectiveness of monitoring and forecasting.

At the same time, solutions based on IoT, edge/cloud computing, and multi-agent systems are being developed to increase scalability, reduce costs, and shorten response times. Another clear trend is the automation of data processing and the creation of open-source tools, which accelerates the transfer of new methods into seismic practice.

IoT technologies, edge and cloud computing, and multi-agent systems are also being developed to increase scalability, reduce costs, and shorten response times. An important trend is the automation of data processing and the creation of open-source tools, which accelerates the adaptation of new methods in seismic practice.

### 3.4. Fires

The fewest publications were in the area of fires, which nevertheless remains an important subject of research. Between 2015 and 2024, 12 articles on this topic were published.

Ref. [103] describes the design and implementation of an automatic forest fire detection system that combines Arduino technology, smoke and flame sensors, and image processing algorithms, enabling rapid fire detection and immediate alerts to forest districts for effective response. Ref. [104] presents an innovative forest fire detection framework that integrates IoT sensors with a CNN model, enabling accurate and rapid real-time fire detection, highlighting the high effectiveness of the model and discussing future challenges related to implementation, scalability, and ethical aspects. Ref. [105] shows an automatic smoke detection system based on video monitoring and image processing, using smoke detection, tracking of moving objects, and cascade classification via CNNs, resulting in high detection efficiency and a reduction in false alarms. Ref. [106] introduces a multi-sensor network for monitoring and forecasting forest and land fires. The system integrates diverse environmental sensors with machine learning algorithms, enabling highly effective early detection and prediction (93.6%) based on field tests conducted, among others, in Riau Province, Indonesia. Ref. [107] proposes an optimized lightweight OFAN (Optimized Fire Attention Network) designed for efficient real-time fire detection on edge devices. Ref. [108] details a modified deep learning model built upon the Inception-V3 network, optimized for detecting fire and smoke in images. Due to a newly developed optimization function, it achieved superior performance and the lowest false alarm rate compared with previous fire detection approaches. In Ref. [109], the authors examine the use of drones equipped with specialized sensors and deep learning techniques such as YOLO and RCNN for effective monitoring and early forest fire detection, demonstrating promising results in real-time fire identification and tracking. Ref. [110] introduces the Integrated Satellite System (ISS), which combines advanced satellite imaging with the Fire Danger Dynamic Index (FDDI) to rapidly detect and prioritize fires, providing integrated data on active fires and fire risk in near real time, which supports effective management of firefighting interventions and reduces environmental and social impacts. Ref. [111] outlines the design of an advanced cyber–physical system that integrates IoT sensors with autonomous aerial and ground drones for early detection and precise suppression of forest fires, enabling continuous monitoring, autonomous firefighting actions, and enhanced support for response teams. In Ref. [112], the authors develop and evaluate an intelligent system integrating IoT sensors and machine learning algorithms for early fire detection and real-time temperature monitoring in forests, highlighting its effectiveness and challenges related to privacy, scalability, and potential large-scale deployment in environmental protection. Ref. [113] proposes an early forest fire detection and classification system that combines IoT technology with the YOLOv5 model. Through IoT-based signal verification, it reduces false alarms and delivers real-time reports to fire services, outperforming existing methods in classification accuracy. Finally, Ref. [114] introduces a novel fire pixel segmentation approach based on color theory and a probabilistic reference table, which outperforms 12 other techniques in terms of fire detection effectiveness.

The analysis shows that research on fires focuses mainly on the automation of detection and the reduction in false alarms. Deep learning methods (CNN, YOLO, RCNN, Inception-V3) are commonly applied in combination with visual imaging, satellite data, and environmental sensors.

The integration of diverse data sources within IoT systems—enabling real-time alerts and continuous monitoring of environmental parameters such as temperature, smoke, and flame—is of considerable importance. Multi-sensor and hybrid platforms that fuse information from satellites, drones, and ground stations are becoming increasingly prominent.

Another emerging trend is the use of edge devices for real-time fire detection, which enables reliable operation in environments with limited connectivity. Recent studies also highlight ongoing challenges related to scalability, privacy, and the ethical implications of deploying such systems.

The main research areas in the category of fire monitoring are presented in Table 5.

It is worth noting that the literature discussed in the above section does not indicate a single universal “leader” among CNN, YOLO, R-CNN, and Inception-V3, as the choice of architecture depends on the type of task and implementation conditions. However, typical trade-offs are emerging: YOLO is most often chosen in scenarios requiring fast inference (near real time), usually at the expense of slightly lower precision in difficult cases; R-CNN more often offers higher detection accuracy, but is more computationally expensive; CNN/Inception-V3 perform well in classification, but in operational applications often requires supplementation with detection/segmentation. As a result, the literature suggests selecting a model as a compromise between accuracy, speed, and hardware resources.

### 3.5. Summary of Literature Review

To systematize the results of the literature review on the four main areas of risk—floods, air pollution, earthquakes, and fires—a summary table was prepared presenting the key research areas, technologies used, and their practical applications (see Table 6). This allows for a comparison of approaches used in different areas of hazard monitoring, identifying both common solutions and those specific to a given type of phenomenon. On this basis, a foundation is provided for further analysis of research trends and for identifying potential directions for the development of integrated monitoring systems.

In this review, the term “machine learning” encompasses diverse families of methods that differ in their data requirements and interpretability trade-offs. Classic models (e.g., regularized regressions, trees, random forests, gradient boosting) work well with tabular data and moderate sample sizes, often offering better interpretability and stability, but limited ability to learn representations from raw image or signal data. Deep models, including CNNs, are particularly effective at analyzing images and signals, but typically require larger training sets, careful validation, and are less interpretable. Segmentation architectures (e.g., U-Net and variants) dominate in area mapping/segmentation tasks (e.g., flood extent), while detection models (e.g., YOLO/RCNN variants) are chosen when the goal is to locate objects/hotspots. In practice, the choice of model class is a function of the data type, task objective, and implementation requirements (latency, computational resources, interpretability).

In summary, the literature review indicates the dynamic development of digital technologies in monitoring natural and environmental hazards. In each analyzed category, there is a growing importance of artificial intelligence, IoT technologies, the fusion of data from multiple sources, and the automation of detection and forecasting processes. Advances in satellite remote sensing, the development of low-cost sensors, and the integration of real-time systems significantly improve the effectiveness of early warning and crisis management.

## 4. Statistical Overview

Table 7 shows a detailed summary of scientific publications from 2015 to 2019 and 2020 to 2024, with 112 documents selected based on an analysis of the Scopus database. The presented data breakdown takes into account the type of document, subject areas related to threat monitoring, and the digital technologies and research methodologies applied. The analysis is accompanied by the results of the Chi-square test, which serves to assess the statistical significance of the observed differences.

There is a clear change in the type of document between the analyzed periods. The largest increase in the number of publications concerns journal articles (from 11 in 2015–2019 to 67 in 2020–2024), a total of 78 works (69.64% of the entire collection). Conference papers accounted for 28.57% (32 publications), while documents classified as other (e.g., book chapters or reviews) appeared only in 2020–2024. The differences in the distribution of document types are statistically significant (*χ*^2^ = 14.29; *df* = 2; *p* = 0.0).

When analyzing the category of hazard monitoring, the largest number of publications concern floods (43), followed by air pollution (32), earthquakes (26), and fires (12). In each of these subcategories, an increase in the number of publications was observed in the years 2020–2024, but these changes were not statistically significant (*χ*^2^ = 1.78; *df* = 3; *p* = 0.62).

The most commonly used technology was machine learning, which appeared in 46 publications (including 44 in the second half of the decade). This was followed by image processing (43) and IoT technologies (33). The differences between periods in this area are statistically significant (*χ*^2^ = 12.73; *df* = 2; *p* = 0.0), which indicates the growing role of emerging technologies in research on threat monitoring.

In terms of research methodology, experiments dominated (80 publications), accounting for over 71% of the total. Conceptual works (47), case studies (31), and literature reviews (29) also accounted for a significant number. Although increases were recorded in each category between 2020 and 2024, they were not statistically significant (*χ*^2^ = 3.01; *df* = 3; *p* = 0.39). At the same time, a tendency towards more in-depth empirical research and theoretical analysis has been observed.

The results indicate a clear increase in the intensity of scientific research in the field of environmental risk monitoring in the years 2020–2024, which may be the result of growing public awareness, progressive climate change, and the dynamic development of digital technologies. The increase in the number of scientific publications in the form of peer-reviewed articles is particularly noticeable, indicating growing interest among the academic community and an improvement in the quality and maturity of the research conducted. In turn, the increase in the share of technologies such as machine learning and IoT technologies indicates the growing penetration of artificial intelligence and automation solutions into research areas related to prevention and early warning. Importantly, these differences are statistically confirmed in terms of document type and technologies applied, which gives them greater credibility. The lack of significant differences in the types of threats and research methods indicates a kind of stability in research topics and the balanced development of different methodological approaches, which provides a foundation for further interdisciplinary analysis.

### 4.1. Document Typology

**Figure 3:**
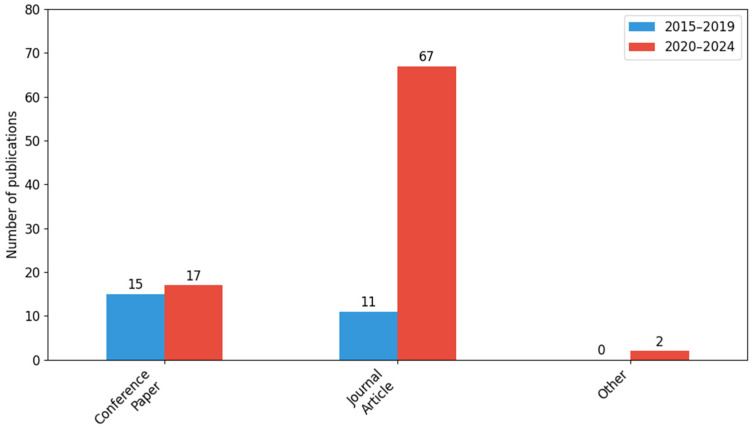
Number of publications in 2015–2019 and 2020–2024 by document type.

Figure 3 shows a comparison of the number of publications by document type in two five-year periods: 2015–2019 and 2020–2024. The comparison includes three categories: conference papers, scientific articles, and other types of documents.

The largest increase in the number of publications was observed in the case of journal articles: from 11 publications in 2015–2019 to 67 in 2020–2024. This increase may indicate the growing importance and maturity of the research topic, which more often takes the form of peer-reviewed papers in scientific journals than conference materials.

Conference papers remained stable, with 15 publications in the first period and 17 in the second. This shows that conferences continue to be an important forum for the exchange of knowledge, but (it should be emphasized) no clear upward trend was observed there.

The category of other documents (e.g., book chapters, reviews) was marginal: only two publications appeared in 2020–2024, with no activity in this area in 2015–2019.

In terms of percentage share for the entire sample (N = 112), scientific articles constitute the dominant (69.64%), conference papers accounted for 28.57%, and other documents totaled 1.79%.

These data confirm the growing professionalism and formalization of research in this area, which is reflected in the significant increase in the number of publications in renowned scientific journals over the last five years.

The data indicate significant shifts in the publication structure, particularly evident in the category of journal articles. Such a marked increase reflects the maturation of this research area and increased interest among the scientific community in the topic of threat monitoring using modern technologies. Conference papers remained at a similar level in both periods, which indicates their (still) important function as a platform for presenting preliminary research results, although no dynamic growth was recorded there. The ‘Other’ category was of marginal importance; only two publications appeared in 2020–2024, which may be due to the fact that the scientific community chooses more formal channels of knowledge distribution. This trend clearly demonstrates the growing professionalization of research in the field of threat monitoring and digital technologies, as well as their increasingly frequent publication in peer-reviewed scientific journals, which translates into greater recognition and quality of studies.

### 4.2. Hazard Monitoring in 2015–2019 and 2020–2024

**Figure 4:**
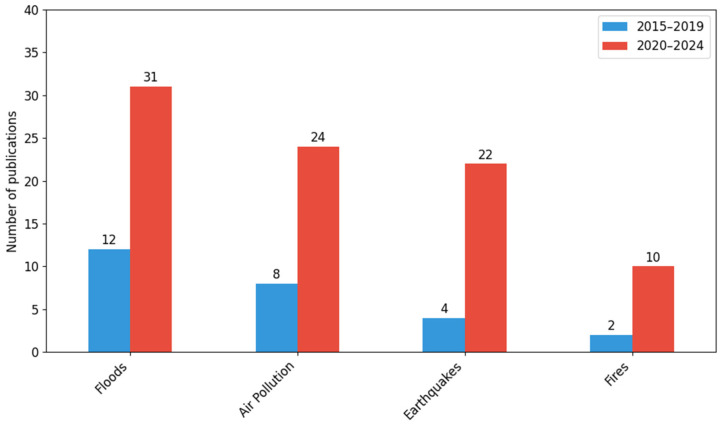
Number of publications in subject area analyzed for 2015–2019 and 2020–2024.

Figure 4 shows the number of publications in four thematic categories related to the monitoring of natural and environmental hazards: floods, air pollution, earthquakes, and fires. The data are broken down into two five-year periods: 2015–2019 and 2020–2024.

In all analyzed categories, there has been a noticeable increase in the number of publications in recent years. The largest number of papers was recorded in the field of flood monitoring, which increased from 12 in 2015–2019 to 31 in 2020–2024.

There was also a significant increase in the category of air pollution, from 8 to 24 publications. Another significant increase can be seen in the area of earthquakes, where the number of publications rose from 4 to 22. In the case of fires, the increase was from 2 to 10 publications.

Despite a clear increase in the number of publications in each category, the Chi-square test (*χ*^2^ = 1.78; *df* = 3; *p* = 0.62) does not indicate the statistical significance of these changes. Although no statistically significant differences were observed (*χ^2^* test, *p* = 0.62), the distribution of publications suggests descriptive tendencies in the representation of hazard monitoring categories over time. These tendencies should be interpreted with caution due to small sample sizes in some categories, particularly fires in the 2015–2019 period.

An increase in the number of publications was recorded in all categories, reflecting the growing importance of research into the effects of climate change and the need to develop early warning systems. The most visible intensification concerns flood monitoring, establishing this topic as a key research area. The results concerning air pollution correspond to the global discussion on public health and the development of urban sensor networks. In the case of the increased number of studies on earthquakes, this indicates the growing use of new seismic detection methods. The topic of fires, although still the least represented, is linked to more frequent extreme forest fires around the world.

### 4.3. Digital Technologies Applied (2015–2024)

**Figure 5:**
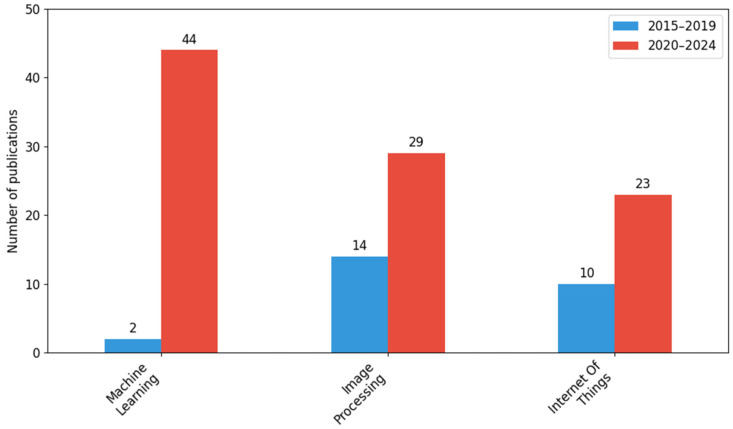
Number of publications classified by digital technology for 2015–2019 and 2020–2024.

Figure 5 shows the number of scientific publications in three categories of digital technologies, machine learning, image processing, and IoT, divided into two five-year periods: 2015–2019 and 2020–2024.

During the analyzed period, there was a noticeable increase in the use of modern digital technologies in research related to threat monitoring. The largest increase was recorded in machine learning, with the number of publications rising from just 2 in the first period to as many as 44 in the second.

In the image processing category, the number of publications increased from 14 to 29, while IoT, although still developing, shows moderate growth—from 10 publications in 2015–2019 to 23 in 2020–2024.

The results of the Chi-square test (*χ*^2^ = 12.73; *df* = 2; *p* = 0.0) indicate statistically significant differences in the distribution of the number of publications between the two periods analyzed.

During the analyzed period, a clear increase in the use of digital technologies in hazard monitoring research was observed, with machine learning developing most dynamically and becoming the dominant analytical tool in the last five years. Image processing maintains a stable position, while IoT shows moderate but consistent growth. Statistically significant differences in the number of publications indicate a real change in the direction of intensifying the use of modern technologies in environmental hazard analysis, reflecting global trends in interdisciplinary research and the development of tools that automate hazard detection and response.

### 4.4. Research Methodologies Applied (2015–2024)

**Figure 6:**
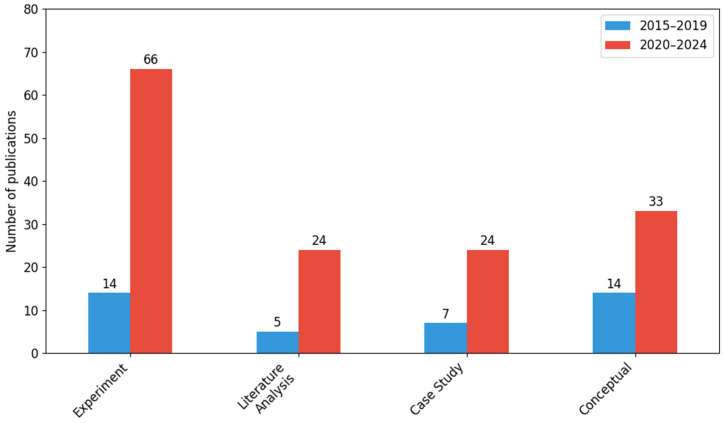
Number of publications classified by research methodology.

Figure 6 shows the number of publications by research methodology used in the two periods analyzed: 2015–2019 and 2020–2024. Four main research approaches were considered: experiment, literature review, case study, and conceptual approach.

The experimental method clearly dominates in the analyzed material, with its use increasing from 14 publications in the first period to 66 in the second, which translates into a total of 80 works (71.43% of all analyzed). Literature analysis also gained in importance, increasing from 5 to 24 publications (380%). This type of methodology was mainly applied in review studies aimed at summarizing the state of the art and identifying research gaps.

Case studies accounted for a total of 31 publications (7 in 2015–2019 and 24 in 2020–2024). The conceptual approach, on the other hand, included mathematical modeling, computer simulations (e.g., in the Matlab environment), and theoretical research. In this case, the number of publications increased from 14 to 33 (136%), which translates into a total of 47 (41.96% of all works).

Despite the apparent increase in the number of publications in each category, the Chi-square test (*χ*^2^ = 3.01; *df* = 3; *p* = 0.39) did not show statistical significance.

The analysis shows that in recent years there has been a marked increase in interest in various research methodologies in the field of threat monitoring. The largest increase was in experimental research, which is the dominant form in the entire collection of publications, indicating a growing trend towards empirical research and the implementation of specific technological solutions. A noticeable increase in the number of literature reviews and conceptual approaches reflects greater involvement of the scientific community in developing theoretical models and summarizing scientific achievements. Despite the increase in the number of publications in all four categories, no statistically significant differences were found, which indicates a relatively even development of individual methodological approaches in the analyzed time period.

### 4.5. Geographical Distribution of Publications (2015–2024)

The highest scientific activity was recorded in China (22 publications, which constitute approximately 20% of the total collection). The number of papers increased from 4 to 18 (an increase of ≈350%). The next country was the United States (20 publications, which constitute approximately 18%) with an increase from 2 to 18 (≈800%), followed by India (18 publications; approximately 16%) with an increase of ≈700% (from 2 to 16). In practice, the countries indicated account for over 53% of all analyzed works (Table 8).

Another group is composed of European countries: Italy (11 publications; ≈10%), Great Britain (10; ≈9%), and Germany (6; ≈5%). Each of these countries saw at least a twofold increase in the number of publications, with the most dynamic growth occurring in Italy (from 2 to 9; ≈+350%).

In the case of South Korea (7 publications), the increase was ≈500% (from 1 to 6). Canada and Japan only appeared in the period 2020–2024 (with 5 publications each). France, on the other hand, maintained a constant number of publications (2 in each period), while in Spain, the number of publications increased from 1 to 3 (a 200% increase).

The category ‘Other’, which includes 11 other countries, experienced an increase from 7 to 12 publications (≈71%). Although the share of a single country in this group does not exceed ≈3% of the total, the overall activity indicates a growing internationalization of research.

**Figure 7:**
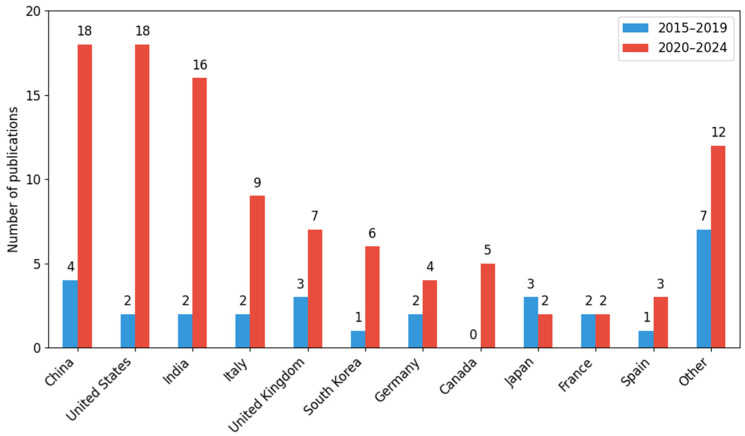
Number of publications in 2015–2019 and 2020–2024 by country.

The geographical analysis of publishing activity presented in Figure 7 shows a clear dominance of Asian and Anglo-Saxon countries in research on technologies applied in hazard monitoring.

The situation was similar in European countries, which may indicate a growing interest in environmental threats in the context of climate change (as evidenced, for example, by legal regulations). South Korea, Japan, and Canada, despite lower absolute numbers of publications, show clear growth in the analyzed area, suggesting that new centers are joining the global scientific discussion. The stable number of publications in France and moderate growth in Spain reflect the maintenance of existing levels of activity rather than an intensification of research. In view of the above, it is important to note the growing internationalization and greater geographical dispersion of research.

The dominance of Asian and Anglo-Saxon countries stems primarily from their high level of technological development, large investments in the Research and Development (R&D) sector, and strategic interest in crisis management and adaptation to climate change. These countries have advanced research infrastructure, a wide network of scientific institutions, and engage in intensive international cooperation, which contributes to high publication productivity in the field of modern hazard monitoring technologies.

### 4.6. The Connection Between Threat Monitoring, Digital Technology, and Research Methodology

When analyzing the data on the links between types of hazards and the digital technologies applied, as well as the research methodology, several correlations can be observed (Table 9). The analysis covers four main categories of hazards: floods, air pollution, earthquakes, and fires. The data are categorized into three groups of variables: digital technologies, research methodology, and the total number of publications.

From the perspective of digital technologies, the most notable correlation is the strong link between image processing and the topic of flooding—34 out of 43 (≈79%) publications in this category. At the same time, image processing was rarely used in research on other hazards, which may indicate the specific nature of visual analysis of hydrological and satellite data. In the case of machine learning, its use was predominant in the context of earthquakes (20 publications; ≈77%) and air pollution (13; ≈41%). The IoT technologies were most frequently applied in the context of air pollution (18 publications; ≈56%), but also in research on fires (6 publications; 50%) and floods (6 publications each; ≈14%). The Chi-square test result (*χ*^2^ = 60.58; *df* = 6; *p* = 0.0) confirms that differences in the use of digital technologies depending on the type of hazard are statistically significant.

In terms of the methods applied, the experimental method dominates in all four hazard categories. For floods, there were 31 publications (≈72%), earthquakes had 21 (≈81%), and air pollution had 19 (≈59%). For fires, there were 9 publications (≈75%).

Literature reviews and case studies were evenly distributed across all categories, with case studies being used more frequently in studies on floods and earthquakes. The conceptual approach was most frequently applied in flood analysis (16 publications; 37%), but also in each of the other categories in similar numbers—13 in the case of air pollution and earthquakes (41% and 50%, respectively), and 5 in the case of fires (42%). The Chi-square test for research methodology (*χ*^2^ = 5.81; *df* = 9; *p* = 0.76) did not reveal any statistically significant differences, suggesting that the choice of research methods was relatively evenly distributed regardless of the type of hazard analyzed.

An analysis of the relationship between the type of hazard, the digital technologies used, and the applied methodological approaches reveals clear thematic and technological patterns. Particularly evident is the strong link between image processing and the theme of flooding due to the specificity of hydrological data requiring visual analyses based on satellite imagery and topographic data, among others. Machine learning, on the other hand, has been particularly frequently used in the context of earthquake and air pollution analysis, due to the large volumes of temporal and sensor data that require advanced predictive analysis. IoT technologies have emerged most frequently in air quality research, reflecting the growing capability to utilize real-time sensor networks.

Experimentation was the dominant research approach in all hazard categories, confirming the strong emphasis on testing and implementing praxis solutions. Although other methods were also present, their share was more equal and less differentiated by hazard type. Statistical significance of differences was only confirmed for digital technologies, while the lack of significance in terms of research methodology suggests that the choice of methods remained relatively universal regardless of the threat context analyzed.

### 4.7. Relationship Between Digital Technologies and the Type of Threat Being Monitored

**Figure 8:**
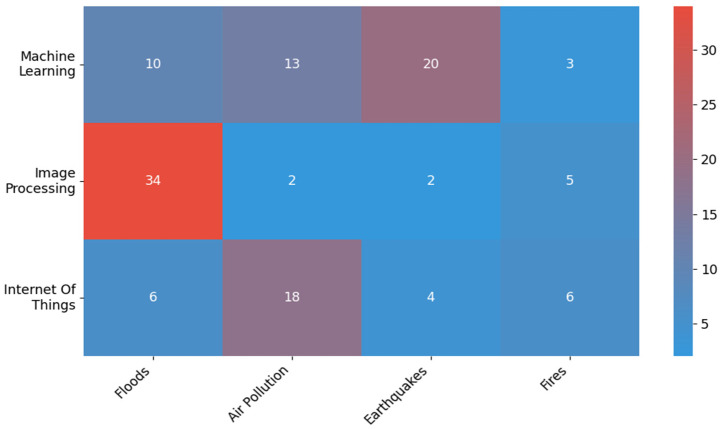
Publications by subject area and hazard categories.

Figure 8 shows the number of publications linking three key digital technologies—machine learning, image processing, and the Internet of Things—to four hazard categories: floods, air pollution, earthquakes, and fires. The result of the Chi-square test (*χ*^2^ = 60.58; *df* = 6; *p* < 0.001) confirms that the distribution of technologies by type of hazard is statistically differentiated.

The strongest relationship was noted for image processing in the flood context: 34 out of 43 publications (≈79%) use visual analysis, highlighting the importance of satellite data and hydrological images in the detection and forecasting of flood events. In other hazards, the share of this technology is marginal (6–8%), indicating its specialized application.

Machine learning dominates earthquake research—20 out of 26 publications (≈77%)—and plays an important role in air pollution (13 out of 32 publications; ≈41%). This result reflects the ability of machine learning models to analyze large, heterogeneous seismic and atmospheric datasets and to detect patterns not visible to the naked eye.

In contrast, IoT technologies were most frequently applied in the context of air pollution (18 of 32 publications; ≈56%) and fires (6 of 12 publications; 50%). Here, IoT sensor networks enable continuous, distributed monitoring of pollutant concentrations or conditions conducive to ignition, which translates into more field research and real-time deployments. In flood studies, IoT appears less frequently (≈14%) and marginally (≈15%) in earthquakes, suggesting the need for further work on sensors that can withstand extreme hydrological and seismic conditions.

The analysis reveals strong specialization in the application of specific digital techniques, depending on the type of hazard being monitored. Image processing shows a clear dominance in flood research, driven by the need to interpret satellite and image data in real time. Machine learning, on the other hand, has found its greatest use in seismic analysis and air pollution monitoring, where large datasets require advanced predictive algorithms. In turn, IoT has been used most frequently in air quality and fire monitoring, owing to the effectiveness of distributed sensor networks in urban and forest environments. The differences in the distribution of digital technology applications are statistically significant, confirming the varying traditional approaches depending on the specific nature of the threat.

### 4.8. Relationship Between Research Methodologies Applied and Type of Threat Being Monitored

**Figure 9:**
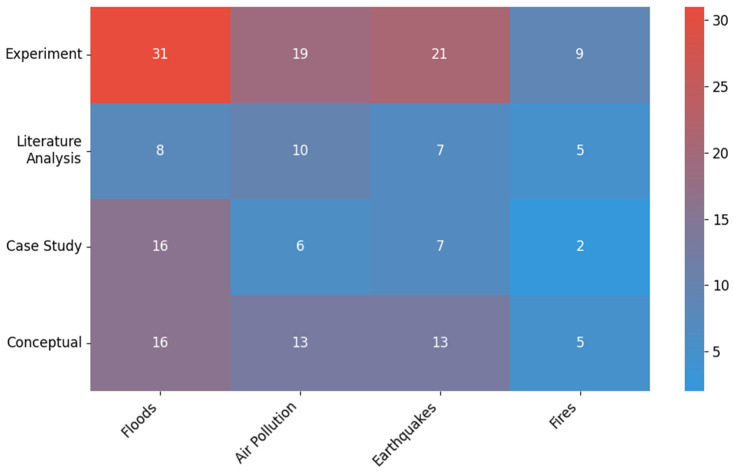
Publications by methodology and hazard categories.

Figure 9 shows the number of publications by four main methodological approaches: experiment, literature analysis, case study, and conceptual approach. This figure is cross-referenced with the four hazard categories: floods, air pollution, earthquakes, and fires. The analysis shows the distribution of research methods according to the type of phenomenon analyzed.

The experimental method is by far the dominant one in all hazard areas analyzed. The largest number of such works concerns floods—31 publications (≈72% of all studies in this category)—confirming a strong orientation towards practical testing of solutions in real conditions, e.g., early warning systems or detection of hydrological phenomena. Similarly, for earthquakes (21 publications; ≈81%) and fires (9 publications; 75%), experimental approaches dominate. A slightly lower proportion is observed for air pollution (19 publications; ≈59%).

Literature analysis was applied in a more balanced way across all categories. It was most frequently used for studies on air pollution (10 publications) and floods (8), while it was least frequently applied for fires (5). Its share ranged from about 18 to 42%. The case study was most frequently used in the analysis of floods (16 publications; ≈37%) and earthquakes (7 publications; ≈27%). Less frequently, the approach was applied in studies of fires (2 publications; ≈17%) and air pollution (6 publications; ≈19%).

Conceptual approaches, including modeling, computer simulations, and conceptual design of technological solutions, were relatively uniformly present in all hazard categories. A total of 13 publications each related to earthquakes and air pollution, while the numbers for floods and fires were 16 and 5, respectively. It is worth noting that, despite the relatively smaller number of conceptual publications in the context of fires, their share (≈42%) is comparable to other hazards.

The observed distribution of methodologies across hazard categories is likely related to the characteristics of the phenomena themselves. Floods and earthquakes, for example, are often studied under real-world conditions, which favors the use of experimental methods. Air pollution, on the other hand, as a more diffuse phenomenon and being more difficult to test directly on a full-system scale, is more often analyzed using literature reviews or conceptual modeling. In turn, fires, due to their unpredictable and hazardous conditions, are relatively less frequently the subject of field experiments, which may explain their lower representation in empirical studies.

The equal use of different research methods—confirmed by the lack of statistical significance in the Chi-square test—is due to the need to combine empirical with theoretical and review approaches in natural and environmental hazard research. The results may therefore reflect not so much a preference for a particular method as the need to align it with the characteristics of the phenomenon under study and the available data.

## 5. Discussion

The results reveal a clear and substantial shift in scientific publications over the period analyzed. In particular, during the second half of the decade, a marked increase was observed, with more than 69% of all publications from the entire study period appearing in those years. This is a result of the increasing maturity of the research topic and the greater recognition within the academic community, as well as the technological opportunities associated with 21st century changes. In contrast, publications in the form of conference papers remained stable, indicating that conferences continue to play an important role in the exchange of scientific knowledge—particularly at the international level—although their influence on the advancement of scientific research appears to be lower than in previous years. Meanwhile, the marginal share of “Other” types of publications (e.g., book chapters) between 2020 and 2024 reflects an increasing preference within the scientific community for more formal and established channels of knowledge dissemination, such as scientific journals. Changes in the structure of the types of papers are important from the point of view of the development of the research discipline itself. The rise in the number of academic articles, including peer-reviewed publications, signifies the increasing professionalization of threat monitoring research. This is because such publications are of greater value in academia and are key to ensuring the reliability and credibility of research findings. The stability in the number of conference papers suggests that conferences remain an important forum for presenting preliminary results and for the exchange of views and experiences among researchers.

### 5.1. Analysis of the Developments in Hazard Monitoring (2020–2024)

An increase in the number of publications on the monitoring of various types of hazards was observed over the period analyzed (especially in the period 2020–2024). The largest increase was observed in the categories of floods, earthquakes, and fires, and although the result (*χ*^2^ = 1.78; *df* = 3; *p* = 0.62) is not statistically significant, these changes may be the result of increasing public awareness, advancing climate change, growing demand for advanced environmental monitoring systems, grants for specific research, and capacity building of research units.

The observed increase in research on floods confirms that this topic remains one of the key research areas. As one of the most dangerous natural hazards, floods require continuous improvement in monitoring and forecasting methods. Meanwhile, the increase in the number of papers on air pollution is a response to the growing health problems associated with air quality, particularly in urban areas. This work often focuses on the development of new monitoring technologies, such as sensor networks. The increase in publications on earthquakes, on the other hand, is due to a growing interest in new methods of seismic detection and an increasing amount of research into improving early warning systems.

### 5.2. Analysis of the Application of Digital Technology

Digital technologies are of particular importance in hazard monitoring research, playing an increasingly important role in environmental hazard analysis and forecasting. The clear leader in this category is machine learning, with the number of publications increasing from 2 in 2015–2019 to 44 in 2020–2024. The results of the Chi-square test (*χ*^2^ = 12.73; *df* = 2; *p* = 0.0) indicate a statistically significant change in the use of the technology. In practice, machine learning—owing to its ability to analyze large datasets quickly and efficiently—has become an invaluable tool for monitoring potential hazards, particularly in the context of earthquakes and air pollution.

The increase in publications in the area of image processing (from 14 to 29) and IoT (from 10 to 23) confirms the growing role of technology in hazard research. Image processing, used mainly for floods, allows for the analysis of satellite and topographic data, which is crucial for the detection and forecasting of such phenomena. IoT, on the other hand, is particularly useful for air pollution studies, as it allows for continuous real-time monitoring, which is important for public health preservation/protection and emergency management.

### 5.3. Analysis of the Research Methodologies Applied

An analysis of the research methods applied indicated that experimentation was the dominant method in the study period, appearing in 66 publications between 2020 and 2024 (approximately 71% of the total). Experimental methods were particularly prominent in research on floods, earthquakes, and fires. This is because such studies are conducted under real-world conditions, which is essential for the development and testing of effective early warning and emergency response systems.

On the other hand, literature analyses and case studies, although representing a smaller portion of the research, have played an important role in developing state-of-the-art reviews and identifying research gaps. In contrast, conceptual approaches, mathematical modeling, and computer simulations were employed to analyze floods and earthquakes, particularly when the goal was to develop new analytical tools.

The results of the analysis carried out indicate that research on environmental hazard monitoring is growing in intensity. The marked increase in the number of scientific publications, especially peer-reviewed articles, is indicative of the growing interest in this research area and the improving quality of research. The increased role of digital technologies, such as machine learning, image processing, and IoT, is a contemporary response to the growing challenges of monitoring increasingly prevalent hazards. However, despite these positive developments, the lack of significant differences in threat types and research methods suggests that there is still a (urgent) need to further develop and improve the methodologies and technologies applied in this area.

The review analysis also indicates that methods based on machine learning and neural networks in most cases contribute to improved detection sensitivity and reduced response times compared to classical approaches based on decision thresholds. At the same time, the effectiveness of the reported solutions remains highly dependent on data availability, spatial and temporal resolution, and the specifics of the analyzed area. The diversity of the datasets and evaluation measures used limits the possibility of direct quantitative comparisons between studies, which means that current work focuses more on the robustness and adaptability of models than on achieving universal levels of accuracy.

The literature also includes attempts to go beyond classic early warning and short-term prediction systems, aiming to identify potential signals preceding catastrophic events, in particular earthquakes. Some studies explore the possibility of using data from outside direct seismic systems, such as changes in ionospheric and electromagnetic parameters or the impact of cosmic factors (e.g., proton streams or solar activity), analyzed using machine learning and neural network methods.

However, it should be emphasized that such approaches remain at the experimental stage, and their repeatability, statistical stability, and operational usefulness in crisis management systems are still the subject of intense scientific debate. For this reason, this review focuses primarily on solutions based on direct data (sensory, imaging, and seismic) that are used in practical monitoring and early warning systems. Nevertheless, the discussed works indicate an important direction for further research on the temporal prediction of extreme events, requiring interdisciplinary cooperation and long-term validation.

## 6. Conclusions

The literature review carried out covered four key research areas related to hazard monitoring: floods, air pollution, earthquakes, and fires.

− Validation/benchmarks: There is a lack of common benchmarks and metrics; limited comparability of results; and difficult selection of methods with real operational advantage.
− Generalizability: There is a dominance of case studies and limited transfer assessment; sensitivity to data shift and data gaps; and decreased reliability in critical applications.
− Near real-time and implementation: There exists rare reporting of latency, infrastructure, and data quality control; incomplete assessment of “from data to decision”; and implementation barriers in warning systems.

The analysis covering publications from 2015 to 2024 showed a significant increase in the number of papers in recent years, indicating a growing interest in the use of modern digital technologies in risk management and environmental protection. In each of the research areas analyzed, a strong trend towards the integration of different data sources and the application of advanced analytical methods, including machine learning and deep learning, to improve the effectiveness of detection, forecasting, and early warning is discernible.

In practice, an analysis of the four main thematic categories—floods, air pollution, earthquakes, and fires—reveals marked differences in the number and dynamics of scientific publications in recent years. The highest number of articles was recorded in the area of flooding, where a total of 43 papers were published, the majority of which were written between 2020 and 2024, indicating a growing interest in the use of modern technologies such as satellite remote sensing (e.g., [3,4,8,9,10,16,18,21,23,24,25,27,28,29,30,38,45]), machine learning (e.g., [5,6,11,14,17,20,28,32,36,37,39,41]), or IoT systems (e.g., [7,12,19,42,44]) in monitoring and forecasting this phenomenon.

In the case of air pollution, 32 articles have been published, mainly focusing on the use of artificial intelligence (e.g., [47,48,49,53,54,57,60,64,67,69,71,77]), predictive modeling, and sensor networks (e.g., [46,51,59,61,66,68,74]) to improve the quality of predictions and manage health risk.

Earthquakes rank third in terms of the number of papers (26 publications), with a significant increase in research in the last five years on the application of deep learning (e.g., [78,79,83,84,85,86,88,90,91,92,93,94,95,96,97,99]) and advanced seismic algorithms (e.g., [81,100,101]) in event detection and localization.

The fewest publications addressed fires (12 papers). However, this area demonstrates a particular focus on developing early warning systems based on IoT (e.g., [103,104,111,112,113]), drones, and deep learning algorithms (e.g., [105,107,108,109]), which shows its significant development potential.

As noted in the Introduction Section, one of the objectives of this article was to identify existing research gaps. Based on the analyses conducted, it is now possible to outline the following:

I. Floods:
  − There is a lack of sufficient validation of flood detection algorithms under real conditions, especially in regions with limited access to satellite data;
  − There is a limited number of studies integrating satellite, hydrological, and IoT data into a single, consistent predictive model;
  − There is insufficient analysis of the impact of climate variability on the effectiveness of current monitoring systems.
II. Air pollutants:
  − There is insufficient research on real-time integration of data from terrestrial and satellite sensor networks;
  − There exists a lack of long-term research on the impact of air quality forecasts on policy decisions and public health.
III. Earthquakes:
  − Little research has been conducted into the use of commonly available mobile devices (smartphones, consumer sensors) as elements of distributed seismic systems;
  − There exists limited adaptation of deep learning models to local conditions, which may reduce the effectiveness of predictions in specific geological regions;
  − There is a lack of consistent global benchmarks to compare the performance of different algorithms.
IV. Fires:
  − Few studies have been conducted on the effectiveness of early detection systems in difficult environmental conditions (fog, smoke from other sources, variable lighting);
  − There is a lack of research into the integration of autonomous firefighting agents (robots, ground-based drones) with AI-based detection systems.

In summary, the development of digital threat monitoring technologies offers significant opportunities to improve environmental and social security. Future research work should focus on building modular and interoperable systems that can integrate data from different sources and handle multiple types of threats simultaneously. Incorporating elements of artificial intelligence, real-time processing, predictive analytics, and mobile technologies can significantly increase the effectiveness and availability of such solutions, helping to better prepare communities for emergencies.

Based on a thorough review of the literature and a correlation analysis, it can be concluded that further research on environmental hazard monitoring should focus on several key technological areas. In the case of floods, further integration of satellite, hydrological, and meteorological data with machine learning models is important, as it can improve the effectiveness of early warning systems. Research on air pollution should develop solutions based on distributed IoT networks and real-time data analysis to support public health management. In the area of earthquakes, advanced seismic signal processing methods and hybrid early warning systems using artificial intelligence remain promising. In fire research, on the other hand, the further development of image processing and spatio-temporal analysis algorithms will play a key role, enabling rapid detection and assessment of the dynamics of the threat.

## Figures and Tables

**Table 1:** Quantitative co-occurrence metrics for primary keywords.

Keyword	Occurrences	Total Link Strength	Avg. Publication Year
Floods	42	50	2020.79
Earthquakes	26	27	2021.77
Air Pollution	25	26	2020.36
Machine Learning	24	26	2020.71
Deep Learning	33	31	2022.09
Image Enhancement	17	21	2021.00
Image Processing	15	18	2019.00
Fires	12	13	2022.08

**Table 2:** A summary of the main research areas in flood monitoring, with examples of technologies and applications.

Research Area	Examples of Technology and Methods	Purpose and Use	Examples of Publications
Satellite data and remote sensing	SAR, optical, hyperspectral, multispectral images	Rapid and accurate detection of flooded areas	[3,4,5,6,8,9,10,16,18,20,21,23,24,25,27,28,29,30,32,33,34,35,36,37,38,40,41,45].
IoT integration	Water level sensors, weather stations, environmental measurements	Early warning, real-time monitoring	[7,12,19,30,39,42,44].
ML and AI	CNN, U-Net, HRNet, random forest, SVM	Image segmentation, water level prognosis	[5,6,11,14,17,18,20,28,30,32,36,37,39,41,42].
Hydrological modeling	Hydrodynamic models, Bayesian optimization, hybrid models	Flood risk assessment, hydrological forecasts	[13,16,21].
New indicators and data fusion	NDWI, MNDWI, SOWI, optical radar data fusion	Improving detection and classification accuracy	[8,9,15,29].
Urban and instant mapping	Segmentation of aerial photographs, analysis of built-up areas	Identification of areas difficult to observe	[7,23,28,33,37].
Crowdsourcing and VGI data	Images from drones and smartphones, user data	Satellite data support, rapid flood localization	[14,26].
Specialist applications	Protection of cultural heritage, monitoring of floodplain vegetation	Analysis of the effects of flooding on facilities and the environment	[25,35,43].

**Table 3:** Main research areas in air pollution monitoring.

Research Area	Examples of Technology/Methods	Purpose/Use	Examples of Publications
Air quality monitoring using IoT and sensor networks	Wireless sensor networks, Arduino/Raspberry Pi microcontrollers, cloud communications	Real-time continuous measurement of air quality, supporting environmental management in cities	[46,51,59,61,66,68,74,77].
Machine learning and artificial intelligence in pollution analysis and forecasting	RF models, LightGBM, CatBoost (Categorical Boosting), YOLO (You Only Look Once), CNN, GAN (Generative Adversarial Network), AQI (Air Quality Index) prediction	Forecasting of pollution levels, high-resolution mapping, identification of emission sources	[47,48,49,53,54,57,60,64,66,67,69,71,77].
Reconstruction and completion of missing data	Graph-based methods, Laplacian interpolation, compressive sampling, data fusion	Improving data quality and consistency in heterogeneous sensor networks	[50,52,55,64,65,73].
Calibration of low-cost sensors and validation of measurements	PM2.5 calibration procedures, ozone, optimization algorithms	Increasing the accuracy of measurements from low-cost equipment	[54,70].
Mobile measurement platforms	Unmanned aerial vehicles, gas sensors, multispectral and thermal imaging cameras	Monitoring of odor and pollutant emissions in hard-to-reach locations	[56,62].
Smart city systems and data integration	Cyber–physical–social systems, smart mirrors	Integrated air quality management, alerting residents, reducing health risks	[63,66,72,74,75,76].

**Table 4:** Main research areas in earthquake monitoring.

Research Area	Examples of Technology/Methods	Purpose/Use	Examples of Publications
Machine learning and deep learning	CNN, U-Net, ADE-Net2, CubeNet, MagNet, ConvNetQuake, OBSTransformer	Automatic detection, classification, and location of seismic phases	[78,79,80,81,83,84,85,86,88,90,91,92,93,94,95,96,97,99,100].
IoT and distributed systems	Sensor networks, building monitoring, multi-agent systems	Real-time monitoring, integration of various data sources	[19,87,93,101].
Data processing and predictive models	MALMI, wave migration, transfer learning, regression models	Improving the accuracy of location and magnitude estimation	[82,85,86,92,94,97,100].
Ocean bottom seismometers	OBSTransformer, dedicated deep learning models	Detection of P- and S-phases in submarine conditions	[88,92].
Low-energy systems	LoRa, precision sensors	Vibration recording, structural resistance assessment	[87].
Specialized methods and analyses	ULF classifier, iceberg sharing analysis	Early warning, extended applications of seismology	[98,102].

**Table 5:** Main research areas in fire monitoring.

Research Area	Examples of Technology/Methods	Purpose/Use	Examples of Publications
IoT systems and sensors	Arduino, smoke and flame detectors, environmental sensors	Early fire detection, alarm transmission, temperature monitoring	[103,104,106,111,112,113].
Machine learning and deep learning	CNN, YOLO, RCNN, Inception-V3, OFAN	Automatic smoke and fire detection, reduction in false alarms	[104,105,107,108,109,113].
Drones and autonomous systems	Aerial and ground drones with sensors and AI	Real-time fire monitoring and suppression	[109,111].
Satellite systems and risk indices	FDDI, satellite data	Rapid detection and prioritization of fires	[110].
Image segmentation and analysis	Color methods and probability tables	More effective detection of fire outbreaks	[114].

**Table 6:** A summary of the main research areas, technologies used, and their applications in hazard monitoring.

Type of Risk	Key Research Areas	Examples of Technology/Methods	Purpose/Use
Floods	Satellite data and remote sensing; IoT integration; machine learning and AI; hydrological modeling; new indicators and data fusion; urban and rapid mapping; crowdsourcing; specialized applications	SAR, optical, hyperspectral images; water level sensors; CNN, U-Net, HRNet; hydrodynamic models; NDWI, MNDWI, SOWI; data from drones and smartphones	Rapid detection of flooded areas, early warning, risk forecasts, real-time mapping
Air Pollutants	IoT networks and sensor systems; machine learning and AI; satellite remote sensing; data fusion; specialized monitoring; new data acquisition methods; systemic and social aspects	Arduino, Raspberry Pi, 5G; RF, CatBoost, LightGBM; Sentinel-5P, Landsat; UAV with gas sensors; electronic ‘noses’; fog analysis	Air quality monitoring, forecasting, data gap reconstruction, emission control, community engagement
Earthquakes	Machine learning and AI; distributed measurement systems; IoT integration; multi-source data fusion; microseismicity; early warning systems; automation and the use of open-source solutions	CNN, U-Net, OBSTransformer, CubeNet, MagNet; DAS, OBS, large-N networks; edge and cloud computing; QuakeFlow; Python packages	Real-time detection and location, magnitude estimation, structural monitoring, forecasting
Fires	AI-based early detection; IoT and sensor integration; multi-sensor systems; edge computing; data fusion; false alarm reduction; systemic and ethical aspects	CNN, YOLO, RCNN, Inception-V3, OFAN; Arduino; FDDI; satellites; drones; autonomous firefighting robots	Detection of fire outbreaks, rapid alerting, coordination of firefighting operations, minimization of losses

**Table 7:** Publications by year in all categories.

Name	2015–2019	2020–2024	All Years	Share [%]	Chi-Square
Total	26	86	112	100.0	*χ* ^2^
Document type					
Conference paper	15	17	32	28.57	*χ*^2^ = 14.29 (*df* = 2, *p* = 0.0)
Journal article	11	67	78	69.64	
Other	0	2	2	1.79	
Hazard monitoring					
Floods	12	31	43	38.39	*χ*^2^ = 1.78 (*df* = 3, *p* = 0.62)
Air pollution	8	24	32	28.57	
Earthquakes	4	22	26	23.21	
Fires	2	10	12	10.71	
Digital technologies					
Machine learning	2	44	46	41.07	*χ*^2^ = 12.73 (*df* = 2, *p* = 0.0)
Image processing	14	29	43	38.39	
Internet of Things	10	23	33	29.46	
Research methodology					
Experiment	14	66	80	71.43	*χ*^2^ = 3.01 (*df* = 3, *p* = 0.39)
Literature analysis	5	24	29	25.89	
Case study	7	24	31	27.68	
Conceptual	14	33	47	41.96	

**Table 8:** Publications by year and country.

Country	2015–2019	2020–2024	All Years	Share [%]	Chi-Square
All countries	26	86	112	100.0	*χ*^2^ = 14.11 (*df* = 11, *p* = 0.23)
China	4	18	22	19.64	
United States	2	18	20	17.86	
India	2	16	18	16.07	
Italy	2	9	11	9.82	
United Kingdom	3	7	10	8.93	
South Korea	1	6	7	6.25	
Germany	2	4	6	5.36	
Canada	0	5	5	4.46	
Japan	0	5	5	4.46	
France	2	2	4	3.57	
Spain	1	3	4	3.57	
Other	7	12	19	16.96	

**Table 9:** Publications by hazard monitoring in other categories.

Name	Floods	Air Pollution	Earthquakes	Fires	Total	Chi-Square
Total	43	32	26	12	112	*χ* ^2^
Digital technologies						
Machine learning	10	13	20	3	46	*χ*^2^ = 60.58 (*df* = 6, *p* = 0.0)
Image processing	34	2	2	5	43	
Internet of Things	6	18	4	6	33	
Research methodology						
Experiment	31	19	21	9	80	*χ*^2^ = 5.81 (*df* = 9, *p* = 0.76)
Literature analysis	8	10	7	5	29	
Case study	16	6	7	2	31	
Conceptual	16	13	13	5	47	

## Data Availability

Not applicable.
